# Analysis of COMPASS, a New Comprehensive Plasmid Database Revealed Prevalence of Multireplicon and Extensive Diversity of IncF Plasmids

**DOI:** 10.3389/fmicb.2020.00483

**Published:** 2020-03-24

**Authors:** Pierre-Emmanuel Douarre, Ludovic Mallet, Nicolas Radomski, Arnaud Felten, Michel-Yves Mistou

**Affiliations:** ^1^Agence Nationale de Sécurité Sanitaire de l’Alimentation, de l’Environnement et du Travail, Laboratory for Food Safety, Paris, France; ^2^INRAE, MaIAGE, Université Paris-Saclay, Jouy-en-Josas, France

**Keywords:** plasmid database, host range, replicon typing, MOB typing, plasmid mobility, multireplicon, IncF plasmid

## Abstract

Plasmids are genetic elements that enable rapid adaptation and evolution by transferring genes conferring selective advantages to their hosts. Conjugative plasmids are predominantly responsible for the global dissemination of antimicrobial resistance, representing an important threat to global health. As the number of plasmid sequences grows exponentially, it becomes critical to depict the global diversity and decipher the distribution of circulating plasmids in the bacterial community. To this end, we created COMPASS, a novel and comprehensive database compiling 12,084 complete plasmids with associated metadata from 1571 distinct species isolated worldwide over more than 100 years. The curation of the database allowed us to identify identical plasmids across different bacteria revealing mainly intraspecies dissemination and rare cases of horizontal transmission. We outlined and analyzed all relevant features, plasmid properties, host range and characterized their replication and mobilization systems. After an exhaustive comparison of PlasmidFinder and MOB-typer, the MOB-typer-based analysis revealed that the current knowledge embedded in the current typing schemes fails to classify all the plasmid sequences collected in COMPASS. We were able to categorize 6828 and 5229 plasmids by replicon and MOB typing, respectively, mostly associated with *Proteobacteria* and *Firmicutes.* We then searched for the presence of multiple core genes involved in replication and propagation. Our results showed that 2403 plasmids carried multiple replicons that were distributed in 206 bacterial species. The co-integration of replicon types from different incompatibility (Inc) groups is an adaptive mechanism, which plays an important role in plasmid survival and dissemination by extending their host range. Our results highlight the crucial role of IncF alleles (present in 56% of all multireplicons) and revealed that IncH, IncR, and IncU replicons were also frequently carried in multireplicons. Here, we provided a comprehensive picture of the different IncF subtypes by identifying 20 different profiles in 849 IncF multireplicons, which were mostly associated with *Enterobacteriaceae*. These results could provide the basis for a novel IncF plasmid nomenclature based on different allelic profiles.

## Introduction

Plasmids are extrachromosomal fragments of DNA that can replicate with different degrees of autonomy from the host’s replicative proteins and can transfer between bacterial species. They are found in the three domain of life and are widely represented in bacterial species. Plasmids can either be linear or circular, vary in size, and can represent a large proportion of the whole bacterial genome (Smillie et al., 2010; Shintani et al., 2015). These mobile genetic elements have a typical modular structure consisting of a conserved backbone region ensuring their survival or propagation and a variable accessory region that may encode host-beneficial traits (Garcillán-Barcia et al., 2011). The acquisition or deletion of adaptive modules from different phylogenetic origins begets enormous diversity among plasmids (de Toro et al., 2014; Pesesky et al., 2019). Plasmids are a keystone of horizontal gene transfer, facilitating the rapid evolution and adaptation of their hosts under changing environmental conditions. Plasmids contribute to the epidemic propagation of antibiotic resistance genes in bacterial pathogens and the dissemination of multidrug resistant plasmids is an increasing threat for modern medicine (Carattoli, 2013; von Wintersdorff et al., 2016). Some plasmids can be transferred between different bacterial species from diverse taxonomic groups and thus have a significant ecological impact. Understanding the genetic plasticity and transmission routes of plasmids is crucial in fighting against antimicrobial resistant pathogens.

The classification of plasmids using typing schemes based on replication or mobilization apparatus (replicon or MOB typing) is helpful to describe plasmid diversity but their lack of resolution limits their use for epidemiological studies (Orlek et al., 2017c).

The term “replicon” can refer to any DNA sequence that is capable of replicating as a unit such as a plasmid or can refer more precisely, like in this study to specific regions or genes encoding functions that enable the replication (Carattoli et al., 2014). Plasmids sharing one or more elements of the plasmid replication (same origin of replication or same replicon) or partitioning systems cannot stably coexist in a cell together and are defined as incompatible. Competition for replication factors also lead to competition between plasmids; meaning that plasmid having growth advantages rapidly outgrow other plasmids in the cell (Velappan et al., 2007). Traditionally, replicon typing classifies plasmids according to incompatibility (Inc) groups.

Plasmid mobility is determined by the propagation module that contains the mobilization system (relaxase and origin of transfer—*oriT*) and the mating pore formation (MPF) system. During the conjugation process, the relaxase recognizes the *oriT* (a short sequence of tens to hundreds of base pairs) and cuts the plasmid DNA at a conserved *nic* site to initiate the transfer into recipient cells while the MPF build the mating channel (Smillie et al., 2010). Thanks to their relaxase-specific properties, it is possible to assign a MOB-group to *oriT* sequences. The presence/absence of these three components allows the prediction of the plasmid mobility (Robertson and Nash, 2018).

While Whole Genome Sequencing offers a high-resolution method for studying the genetic structure and variability of plasmids, full exploitation of this massively available data is still a hurdle. Identifying and tracking circulating plasmids heavily relies on the ability to accurately assemble their whole genome sequences, which remains particularly challenging from current short reads mainly due to the presence of numerous repetitive elements, especially in large plasmids (>50 kb) (Arredondo-Alonso et al., 2017). Even though several bioinformatics tools can be applied to reconstruct plasmid sequences from short reads, a contiguous assembly is still difficult to obtain (Zhou and Xu, 2010; Lanza et al., 2014; Antipov et al., 2016; Rozov et al., 2017). This pitfall can be overcome by using long read sequencing technology such as *Oxford Nanopore* or *PacBio Technologies* (Hoffmann et al., 2017; Margos et al., 2017; Li et al., 2018).

With the advances of sequencing technologies and bioinformatics tools, the number of plasmid sequences available in public databases is growing rapidly. However, comparing newly sequenced plasmids with existing resources is cumbersome because of partial and misannotated sequences as well as a paucity in bioinformatic tools to explore the large amount of available data. The creation of a curated and comprehensive complete plasmid database is critical to integrate and elicit understanding of the available data. The first attempt was made by Orlek et al. (2017a), but the database was restricted to complete plasmids belonging to the *Enterobacteriaceae* family (Orlek et al., 2017a). More recently, a database containing all the complete plasmids from the NCBI’s RefSeq database was integrated in the visualization and exploration tool pATLAS to facilitate the identification of plasmid from assemblies (Jesus et al., 2019). A similar database called PLSDB was also implemented in a user friendly and interactive web server, which offers the possibility to upload new sequences and query against the database (Galata et al., 2019). Both PLSDB and pATLAS databases allow the browsing and filtering of plasmids with their associated metadata and annotations. There are also bioinformatics tools that use plasmid databases as a reference for plasmid detection and identification. For example, the dataset that was created by Robertson and Nash (2018) and implemented in the MOB-suite software to reconstruct draft genome assemblies using known plasmid sequences.

In the present paper, we unveil the construction and the curation of a Comprehensive and Complete Plasmid Database we named “COMPASS.” This new database constituted of 12 084 plasmid sequences expands previously described resources and provides a full description of its content in terms of relevant properties (size, taxonomy, geographical origin, and year of isolation). With the objective of getting a global overview of the diversity of circulating plasmids, we characterized the database by *in silico* typing based on replication and mobilization systems and predicted the mobility from the analysis of dissemination markers. The distribution in the bacterial community was carefully examined to highlight particular features and specific associations between core genes and the plasmid host range.

## Materials and Methods

### Creation and Curation of the COMPASS Database

Complete bacterial plasmid accessions were retrieved from the NCBI nucleotide database on 17 June 2018 using advanced optimized filters in the entrez query to exclude incomplete and misannotated plasmids. More precisely, keywords such as “plasmid,” “bacteria,” and “complete” were used in the initial query to retrieve complete plasmid records. Specific terms in the title were filtered to remove plasmid “genes, cds, origin of replication or region,” and other words such as “integron, transposon, operon, and phage” were applied to get rid of mobile genetic elements other than plasmids. Fasta and GenBank files associated with all plasmid accessions were downloaded from the NCBI database. A python script was developed to extract metadata from the GenBank files and to curate the database as described below. Duplicated records were first identified based on specific metadata. All the plasmids with the same size, topology, taxonomy, and header description were considered identical. Duplicated records were removed keeping preferentially the most recent version and RefSeq entry. Duplicated sequences sharing 100% identity and coverage were then identified using the clustering tool CD-HIT-EST (Li and Godzik, 2006). This step allowed the identification of clusters constituted of identical plasmids. One plasmid sequence was kept (as a reference) in the database while the other records were discarded. Finally, putative chromosomal sequences mislabeled as plasmids were identified by performing *in silico* Ribosomal Multilocus Sequence Typing (rMLST) analysis (Jolley et al., 2012). rMLST is an approach which indexes variation of the 53 genes encoding the bacterial ribosome protein subunits (*rps* genes) to identify rapidly the phylogenetic position of any bacterial sequence. The 53 *rps* genes, downloaded from PubMLST^1^ (12 February 2019) were sought in the plasmid records using BLASTn and hits with at least 95% identity and 95% coverage were considered potential matches (Jolley et al., 2012). The scripts and database of COMPASS developed in the present study can be found in the following GitHub repository: https://github.com/itsmeludo/COMPASS.

### Plasmid Typing and Predicted Mobility

*In silico* predictions of the replication gene (replicon), mobilization protein (relaxase), mate-pair formation (MPF), and the origin of transfer (*oriT*) types were obtained using the MOB-typer module from the MOB-suite package^2^ (Robertson and Nash, 2018). Replicon, relaxase, MPF, and *oriT* sequences were identified by BLAST using default parameters against reference databases that Robertson and Nash built for the MOB-typer tool. These databases contained 2481 plasmid-derived replication genes that corresponded to 1683 replicon types, 916 relaxase sequences classified into six MOB families (MOB_F_, MOB_H_, MOB_Q_, MOB_C_, MOB_P_, and MOB_V_), 2066 MPF proteins clustered in four groups (MPF_I_, MPF_F_, MPF_G_, and MPF_T_), and 502 *oriT* sequences.

The plasmid mobility was predicted based on the presence–absence of the three dissemination markers. A plasmid was labeled as “conjugative” if it carried a relaxase plus an MPF and as “mobilizable” if it contained at least a relaxase or an *oriT* whereas plasmids missing both a relaxase and an *oriT* were classified as “non-mobilizable” (Robertson and Nash, 2018).

Replicon typing was also performed using the conventional *in silico* tool PlasmidFinder (version October/2019)^3^ with recommended parameters (Carattoli et al., 2014) and the results were compared to those obtained with MOB-typer. The replicon content of the MOB-typer database was also compared to the one of PlasmidFinder using the clustering tool CD-HIT-EST-2D (i.e., 100% identity and 100% coverage) to identify specific alleles shared between the two typing resources.

### Visualization and Statistical Analysis

The plasmid size was analyzed with R scripts (R_Core_Team, 2018) and graphically represented with the ggplot2 library (Wickham, 2016). A custom world map showing the distribution and the number of plasmids isolated per country was created using the online tool Gunnmap 2^4^. The exploration of the taxonomical composition of the COMPASS database was performed using the web-based interactive visualization tool Krona (Ondov et al., 2011). The HTML link for the interactive Krona chart can be found in the COMPASS GitHub repository mentioned above. The plasmid content of our database was compared to other plasmid resources (PLSDB, pATLAS, and MOB-suite) by comparing the nucleotide databases 2 by 2 and identifying strictly identical plasmid sequence (i.e., 100% identity and 100% coverage) using the clustering tool CD-HIT-EST-2D. The integrative venny tool (Bardou et al., 2014)^5^ was also used to visualize the number of plasmids shared between COMPASS and the other three databases by comparing the lists of accessions. Two co-occurrence network analyses were performed independently based on the R igraph library to highlight plasmid mobilization in different species and decipher the linkages between replication genes present in multireplicon plasmids. Sankey diagrams were built using the online tool SankeyMATIC^6^ to show particular associations between typing results and taxonomy.

## Results

### Construction and Curation of the COMPASS Database

A total of 22,425 sequences representing complete plasmids were retrieved from the NCBI nucleotide database. Duplicate records were first identified based on metadata; records presenting identical size, topology, taxonomy, and header were considered as duplicates. These filtering steps led to the identification of 9299 duplicated records. Among these, 6329 arose from different databases (RefSeq vs GenBank, e.g., NZ_CP018683.2 vs CP018683), 22 were different versions of the same sequence (updated vs older version (e.g., CP022061.2 vs CP022061.1), and 2948 had different accession numbers (CP004864.1 vs CP007611.1). The list of redundant records removed from the initial list is available in Supplementary Table S1. The remaining 13,126 plasmids records were subjected to the stringent clustering tool CD-HIT-EST to remove strictly identical sequence (i.e., 100% identity and 100% coverage).

In total, 11,237 singletons (i.e., unique sequences) and 849 clusters containing from two (89%) to 25 identical plasmids were detected during this clustering step (Supplementary Table S2).

The analysis of these clusters through a manual curation revealed that for 547 clusters, the plasmids within each cluster were associated with the same bacterial strains. These duplicated records were not detected by the first curation step because the headers of their sequences were different (e.g., “complete_sequence” vs “complete_genome”; “mega_plasmid” vs “megaplasmid”; “virulence_plasmid_pCP301” vs “plasmid_pCP301”…). On the other hand, 47 clusters were missing information regarding the bacterial strains and could not be categorized. Interestingly, for 234 clusters, plasmids within each cluster were associated with the same species but different strains, emphasizing the strong species–specific plasmid dissemination. In contrast, rare cases of horizontal transmission between different species were also observed in 21 clusters. These clusters were represented in Supplementary Figure S1, where nodes are bacterial species and edges indicate the sharing of identical plasmid. The result highlights that most of the subgraphs (*n* = 19) group together bacterial species belonging to the same family. For example, the 33 kb plasmid of the cluster #7470 that was isolated in the United States from three different Enterobacteria (*Enterobacter hormaechei*, *Klebsiella oxytoca*, and *Enterobacter cloacae*) over 3 years (2010–2012). However, rare transfers between more distantly related organisms can also occur; like the 46.5 kb plasmid found in two bacteria from different orders (*Acinetobacter lwoffii* and *Klebsiella aerogenes*, cluster #6503). One reference plasmid was kept within each 849 clusters for downstream analyses and 1040 redundant sequences were discarded (Supplementary Tables S1, S2). Finally, chromosomal sequences were identified based on the number of RPS genes present. Out of the 12,086 plasmids, 108 contained less than 10 RPS genes while two records NZ_CP014062.1 and NZ_CP022019.1 had 55 and 54 exact matches with RPS genes of the rMLST database. The predicted taxa of these two records were *Salmonella enterica* and *Pseudomonas putida*, respectively. These two chromosomal sequences were excluded from the database that finally contains 12,084 plasmids. These two records have since been updated on the current GenBank database as “chromosome, complete genome,” which confirmed the present rMLST results.

### Comparison of Plasmid Databases

Our COMPASS database shared 11,374 plasmids (94%) with PLSDB (Galata et al., 2019), 9873 plasmids (82%) with pATLAS (Jesus et al., 2019), and 8271 plasmids (68%) with the MOB-suite database, respectively (Robertson and Nash, 2018) (Supplementary Figure S2 and Supplementary Table S3). Altogether, 6440 plasmids were shared between COMPASS, and the other three databases. The content of the three published databases was also compared with COMPASS to confirm the overlap between plasmid resources. MOB-suite, PLSDB, and pATLAS shared 11,610 (96%), 11,380 (83%), and 10,204 (77%) plasmids with COMPASS, respectively (Supplementary Table S3). The differences observed among the number of plasmids shared between two specific databases (for example, COMPASS versus MOB-suite and MOB-suite versus COMPASS) revealed the presence of duplicates. The presence of identical sequences within a database was indeed confirmed (by running CD-HIT-EST 100%) for MOB-suite (*n* = 3500), pATLAS (*n* = 381), and PLSDB (*n* = 7) but not for COMPASS. The list of duplicated records is available in Supplementary Table S3. The differences in composition could be explained by the construction and curation steps that were specific to each database. The keywords used for the original query to download plasmid records and the inclusion/exclusion criteria in the filtering steps were diverse. Duplicated records were also removed differently; redundant sequences were identified based on pair-wise distances created by the program mash for the PLSDB database while Robertson and Nash removed Refseq accessions, which contained the accession of another records (Supplementary Methods of the MOB-suite paper).

### Description of the COMPASS Database

In order to generate a comprehensive database, all the metadata available from the GenBank files were extracted for each plasmid and carefully examined. The information regarding the size, the topology, and the complete lineage was available for all the plasmids whereas the other metadata were partially available. Relevant metadata describing the host range and transmission route are presented below. All the other metadata can be found in Supplementary Table S4.

**FIGURE 1:**
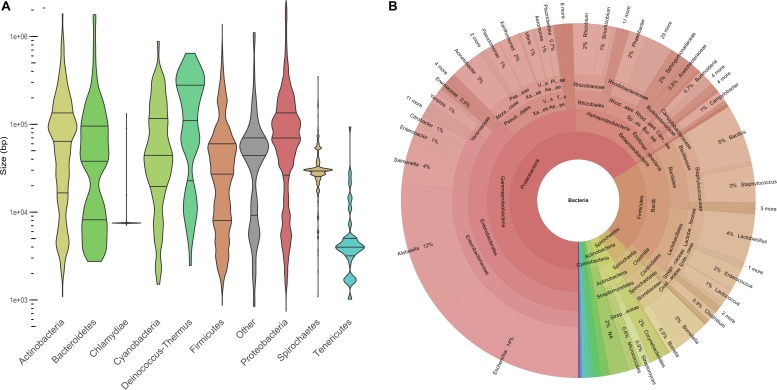
Description of the COMPASS database. **(A)** Violin plot displaying plasmid size distribution (log_10_) among the main phyla (*n* = 12,084). The phylum entitled “Other” is composed of 57 plasmids from 12 minority phyla (*n* < 25) and 129 plasmids missing taxonomy data. **(B)** Krona plot showing the compositions of taxa and taxonomic ranks (*n* = 12,084).

Plasmids ranged in size from 744 to 2,555,069 bp, with a median at 53,206 bp. The plasmid size distribution varied among the different phyla (Figure 1A). Large plasmids occurred more frequently in *Deinococcus-Thermus*, *Proteobacteria*, and *Actinobacteria* than the rest of the phyla (Figure 1A). The highest frequency of large plasmids was observed for *Deinococcus-Thermus*, while the largest plasmids were found in *Proteobacteria*. Indeed, 132 plasmids were larger than 1 Mb and 72% of these “megaplasmids” were isolated from *alphaproteobacteria*. The plasmids isolated from *Chlamydia* and *Tenericutes* were overall smaller than the rest of the plasmids with a median at 7504 and 3990 bp, respectively. With regard to the first and third quartile close to the median, the size of the chlamydial plasmids was very homogenous. Indeed, 70 chlamydial plasmids (92% of the phylum) exhibited a size ranging between 7415 and 7553 bp. This group included the plasmids pCTA, pCHL1, pSW2 from *Chlamydia trachomatis*, pMoPn from *Chlamydia muridarum*, and pCpGP1 from *Chlamydia caviae* (Zhong, 2017).

The metadata “Country” locating the isolation was available for 7096 plasmids (59%), tallying 126 countries with a frequency ranging from one plasmid collected in 21 different countries to 1703 plasmids collected in the United States (Supplementary Figure S3).

The metadata “Collection date” was obtained for 5819 plasmids (48%). Plasmids sequences were obtained from isolates dating from 1884 to 2018 with an exponential increase after 2000, accounting for 85% of all plasmid present in the COMPASS database (Supplementary Figure S3).

The vast majority (94%) of the plasmids were circular and among the 665 plasmids that were linear, 48% were isolated from bacteria belonging to the *Borreliaceae* family.

Our plasmid database covered 1571 distinct bacterial species, 443 genera, 189 families, 93 orders, 37 classes, and 21 phyla. *Proteobacteria* (63%) and *Firmicutes* (21%) were the most represented phyla and 32% of the bacterial species (*n* = 3963) belonged to the *Enterobacteriaceae* family (Figure 1B). Overall, *Escherichia coli* (*n* = 1672), *Klebsiella pneumoniae* (*n* = 1053), and *S. enterica* (*n* = 477) plasmids were the most prevalent species. The taxonomy distribution of COMPASS was compared at the phylum level to that of the PLSDB database and we did not find a significant difference between both distributions (Kolmogorov–Smirnov test, *p*-value = 0.997).

### Replicon Typing

Using MOB-typer, the presence of at least one replicon was detected for 9231 plasmids (76.4%) (Supplementary Table S5). Due to biases related to the constitutions of GenBank and replicon databases, the number of plasmids encoding replicons varied regarding the taxonomy (Supplementary Figure S4). For instance, 81.8% of the plasmids associated with *Firmicutes* and *Proteobacteria* contained at least one replicon whereas only 29.6 and 18.2% of plasmids from *Cyanobacteria* and *Spirochaetes* matched the replicon database, respectively. These observations stressed the knowledge gap of plasmid biology associated with less represented taxa in sequence databases. The number of replicons detected within a plasmid varied from a single gene identified in 6828 plasmids to seven replicons identified in a 446 kb plasmid isolated from *Raoultella ornithinolytica* (Supplementary Figure S5). Interestingly, the amount of replicons within a plasmid does not correlate with the plasmid size (Pearson test: rho = -2%, *p*-value = 6.8 × 10^–3^); meaning that big plasmids do not necessarily possess several replication genes.

**FIGURE 2:**
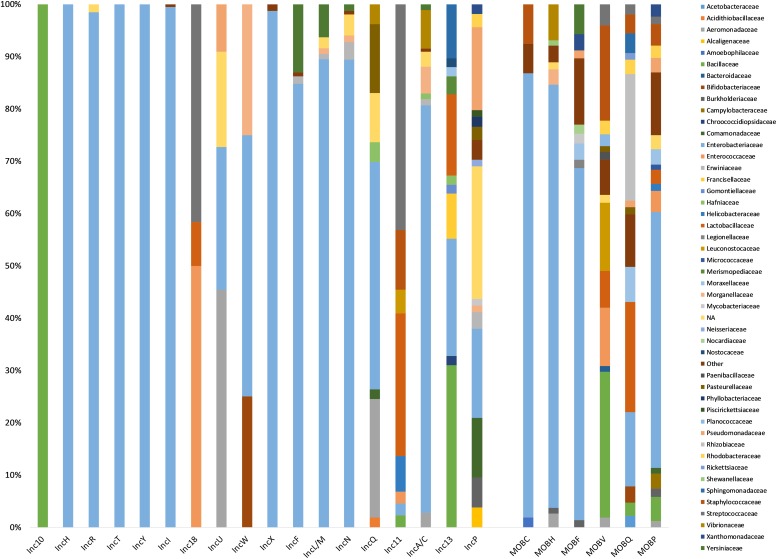
Host range distribution. Percentage of bacterial families in known replicon types (2469 plasmids) and MOB types (5229 plasmids). Bacterial families accounting for less than 1% within replicon types were represented together in the family “Other.”

Among the 6828 plasmids specifically typed by the presence of a single replicon, 2469 plasmids harbored a known replicon from the ColRNAI family (*n* = 639) or were associated with a known Inc group (*n* = 1830) while 4359 plasmids carried a replicon that did not belong to a recognized family or Inc group. These plasmids were associated with 1436 replicon clusters from the MOB-typer database. While 19 Inc groups were identified, plasmids belonging to Inc10 (*n* = 2), IncT (*n* = 1), and IncW (*n* = 4) were rare (Table 1). The most frequently assigned Inc groups by MOB-typer were IncI (362 records) followed by IncX (309 records), IncF (also described as F-type replicons) (269 records), IncA/C (176 records), and IncP (158 records). The plasmids harboring a replicon from the Inc10, Inc18, IncF, IncH, IncI and IncL/M, IncR, IncT, IncU, IncW, IncX, and IncY were associated with a limited host range (less than five bacterial families) while ColRNAI, Inc11, Inc13, IncA/C, IncN, IncP, and IncQ plasmids were present in a broad host range (BHR) (14, 8, 11, 5, 20, and 7 bacterial families, respectively) (Figure 2). The IncL/M family has now been split into IncL, IncM1, and IncM2 subtypes (Carattoli et al., 2015). In addition, the IncA/C group have recently been shown to be compatible and are now distinguished to IncA and IncC (Ambrose et al., 2018). However, the MOB-typer database does not differentiate between the subtypes within these two groups and therefore our analysis did not take these new distinctions into account.

**TABLE 1:** Summary of plasmid replicon type features.

Replicon type	No. of plasmids	Min (bp)	Max (bp)	Median (bp)	No. of families	Host range	Major family host (%)	Predicted mobility (%)	No. of plasmids	No. of relaxases	Major MOB type (%)
ColRNAI	639	1308	51,662	5202	14	BHR	*Enterobacteriaceae* (81.69)	Mobilizable (80.13)	302	5	MOB_P_ (49.01)
Inc10	2	1742	1813	1778	1	Narrow	*Bacillaceae* (100)	Non-mobilizable (100)	0	NA	NA
Inc11	44	1278	15,360	3908	8	BHR	*Streptococcaceae* (43.18)	Non-mobilizable (54.55)	20	2	MOB_V_ (55)
Inc13	58	1643	11,801	3300	11	BHR	*Bacillaceae* (31.03)	Non-mobilizable (56.90)	22	1	MOB_V_ (100)
Inc18	12	5842	70,706	26,725	3	Narrow	*Enterococcaceae* (50)	Non-mobilizable (58.33)	5	1	MOB_V_ (100)
IncA/C	176	3645	233,057	158,754	8	BHR	*Enterobacteriaceae* (77.84)	Conjugative (92.61)	160	2	MOB_H_ (98.75)
IncF*	1118	19,788	416,444	109,349	4	Narrow	*Enterobacteriaceae* (94)	Conjugative (68.25)	861	4	MOB_F_ (82.11)
IncH	19	78,444	223,698	97,393	1	Narrow	*Enterobacteriaceae* (100)	Non-mobilizable (63.16)	7	2	MOB_P_ (71.43)
IncI	362	9466	207,960	83,635	2	Narrow	*Enterobacteriaceae* (99.45)	Conjugative (93.09)	328	2	MOB_P_ (99.39)
IncL/M	95	46,885	95,855	70,092	4	Narrow	*Enterobacteriaceae* (89.47)	Conjugative (91.58)	87	1	MOB_P_ (100)
IncN	151	20,225	17,4695	54,242	5	BHR	*Enterobacteriaceae* (89.40)	Conjugative (97.35)	147	2	MOB_F_ (97.96)
IncP	158	3269	1,499,175	60,505	20	BHR	*Enterobacteriaceae* (17.09)	Conjugative (76.58)	122	3	MOB_P_ (95.90)
IncQ	53	6388	208,409	8300	7	BHR	*Enterobacteriaceae* (43.40)	Mobilizable (90.57)	48	4	MOB_Q_ (45.83)
IncR	65	18,990	125,961	61,010	1	Narrow	*Enterobacteriaceae* (98.46)	Non-mobilizable (64.62)	223	2	MOB_P_ (95.65)
IncT	1	83,698	83,698	83,698	1	Narrow	*Enterobacteriaceae* (100)	Mobilizable (100)	1	1	MOB_P_ (100)
IncU	22	7995	84,749	38,938	3	Narrow	*Aeromonadaceae* (45.45)	Conjugative (50)	13	1	MOB_P_ (100)
IncW	4	4233	39,924	38,971	3	Narrow	*Enterobacteriaceae* (50)	Conjugative (75)	4	2	MOB_F_ (75)
IncX	309	6464	76,500	43,380	3	Narrow	*Enterobacteriaceae* (98.71)	Conjugative (95.79)	296	2	MOB_P_ (99.66)
IncY	30	56,460	126,046	96,897	1	Narrow	*Enterobacteriaceae* (100)	Non-mobilizable (90)	3	2	MOB_P_ (99.66)

*IncF plasmids include all the plasmids carrying F-type replicons; single replicon (*n* = 269) and multireplicon (*n* = 849).

**FIGURE 3:**
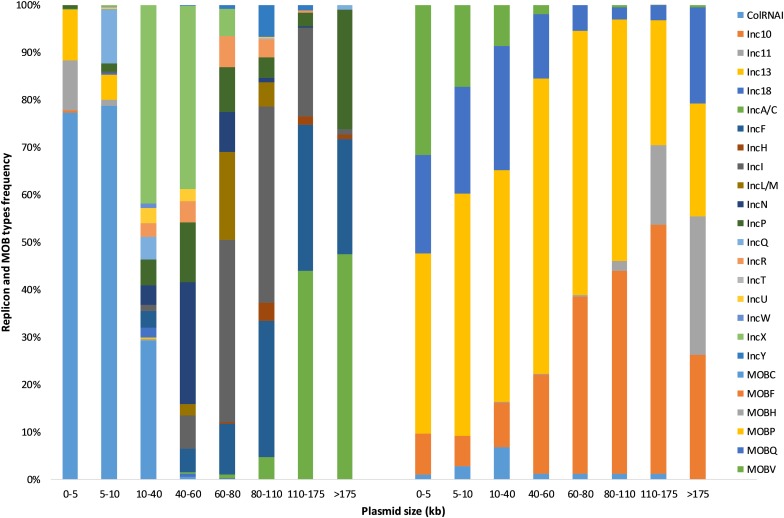
Plasmid size (kb) distribution among the known replicon (*n* = 2469) and MOB types (*n* = 5195) from the COMPASS database.

As expected, Inc grouping schemes corresponded to families for which they were designed to perform plasmid typing. More than 75% of ColRNAI, IncA/C, IncF, IncH, IncI, IncL/M, IncN, IncR, IncT, IncX, and IncY plasmids were associated with *Enterobacteriaceae* family whereas Inc10-11-13-18 replicons most frequently occurred in *Firmicutes* (*Streptococcaceae*, *Bacillaceae*, *Enterococcaceae*) (Table 1 and Figure 2). Replicons clusters were detected in 146 different bacterial families and were the most prevalent in Gram-positive bacteria (393 *Bacillaceae*, 331 *Lactobacillaceae*, and 315 *Staphylococcaceae*). We observed that the plasmid size varied greatly among the different types (Figure 3). More than 80% of the plasmids from the Inc10 (*n* = 2), Inc11 (*n* = 44), Inc13 (*n* = 58), and the ColRNAI (*n* = 639) families were smaller than 10 kb (median at 1778, 3908, 3300, and 5202 bp, respectively) while 80% of the IncA/C (*n* = 176) and IncH (*n* = 19) plasmids were larger than 80 kb (median at 158,754 and 97,393 bp, respectively). Figure 3 also shows that the size of IncP (*n* = 158) and IncF (*n* = 269) plasmids were very diverse (3269 to 1,499,175 and 19,788 to 416,444 bp, respectively).

Multiple replication genes were detected in 2403 plasmids. All the known replicon types from the ColRNAI family and Inc groups were detected except for Inc10, Inc13, and IncW. The most frequent replicons were IncFII (24.5%), IncFIB (*n* = 12.4%), IncFIIA (10.3%), and IncFIA (3.8%) and 56% of the multireplicons (*n* = 1349) carried one of the four IncF alleles. For these 1349 plasmids, a co-occurrence network to visualize the frequently associated replicons highlights the central role of IncF in the multireplicons (Supplementary Figure S6). Overall, multireplicons plasmids were found in 206 bacterial species belonging to 40 bacterial families but were mostly represented in *Enterobacteriaceae* (65.9%) and in Gram-positive bacteria such as *Staphylococcaceae* (7.9%), *Bacillaceae* (3.8%), and *Lactobacillaceae* (3.58%). The combination of replicon types resulted in 539 different profiles of which 138 profiles (1189 plasmids) contained only known replicons. Among these 1189 plasmids, 849 carried only a combination of IncF replicons (20 profiles), 268 plasmids had an IncF that coexisted with another replicon from other Inc groups (84 profiles), and 72 plasmids did not harbor any IncF replicons (37 profiles). The profiles IncU-IncP, IncU-IncX, and IncA/C-IncP were predominant in the plasmids that did not possess IncF while ColRNAI, IncI, IncR, and IncQ were the replicons most frequently associated with IncF. A truncated IncQ replicon inside a resistance region carried by large IncF plasmids has been previously observed by Partridge et al. (2018). In contrast to these 340 plasmids that could not be assigned a typical type, the 849 multireplicons that only contained IncF alleles were categorized as IncF plasmids.

### IncF Plasmids

**FIGURE 4:**
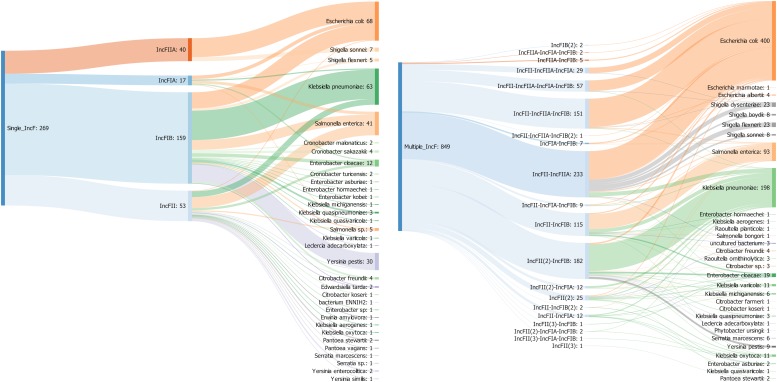
Sankey diagram showing the associations between replicon types and replicon profiles with bacterial species in 1118 IncF plasmids. The number in brackets refers to the copy number of a particular IncF allele.

To gain further insights into the diversity of IncF plasmids, we characterized all the combination of IncF alleles in single and multireplicons and analyzed their host range. Overall, IncF plasmids (*n* = 1118) were detected in 50 bacterial species mainly from *Enterobacteriaceae* (94%) and multireplicons were 3.5 times more prevalent than plasmids carrying a single IncF allele (Figure 4).

Among single replicons, IncFIIA (*n* = 40) and IncFIA (*n* = 17) replicon had a limited host range (present only in two and six species, respectively) compared to IncFIB (*n* = 159) and IncFII (*n* = 53) that were found in 20 and 23 species. IncFIIA and IncFIA occurred significantly more often in *E. coli* whereas IncFIB and IncFII were dominant in *K. pneumoniae* and *S. enterica* respectively (Figure 4). Similar results were previously found by using the original PCR-based replicon typing (PBRT) and the updated replicon sequence typing (RST) schemes (Carattoli et al., 2005; Villa et al., 2010). In contrast to the single replicons, IncFII was detected in almost all the multireplicons (*n* = 833) while IncFIB, IncFIIA, and IncFIA were present in 537, 478, and 131 plasmids, respectively.

The most prevalent profile was IncFII-IncFIIA (*n* = 233), followed by IncFII(2)-IncFIB (*n* = 181) and IncFII-IncFIIA-IncFIB (*n* = 151). The IncF multireplicons associated with the three most prevalent species *E. coli* (47%), *K. pneumoniae* (23%), and *S. enterica* (11%) were very diverse; belonging to 13, 10, and 8 different profiles, respectively. Conversely, some plasmids from other species were associated with a particular type. All the plasmids from *Shigella* (*n* = 62) were linked to the same profile IncFII-IncFIIA and the nine replicons from *Yersinia pestis* belonged to the IncFII(2)-IncFIB profile (Figure 4).

### Comparison Between MOB-Typer and PlasmidFinder

Using the conventional tool PlasmidFinder, replicons were only detected in 4931 plasmids (40%), of which 3349 have been assigned to specific types (Supplementary Table S6). The MOB-typer results of the 6828 plasmids associated with single replicons as well as the 849 IncF multireplicons were compared to the replicon types obtained with PlasmidFinder (Supplementary Table S6). We found a high concordance (>90% match) between the known replicon types identified by MOB-typer and those detected by PlasmidFinder for IncA/C, IncI, IncL/M, IncN, IncQ, IncR, IncT, IncX, and IncY plasmids. However, discrepancies were observed for the other Inc groups. The 116 plasmids belonging to Inc10, 11, Inc13, and Inc18 types were either assigned to different types (*n* = 53) or were not detected (*n* = 63) by PlasmidFinder. Similarly, 84% (*n* = 16) of the IncH plasmids were assigned to a different replicon type (p0111). Finally, only 43% (*n* = 68) of the IncP plasmids were confirmed while 8% (*n* = 12) were classified as different types and 49% (*n* = 78) were not detected by PlasmidFinder. Regarding the 1118 plasmids identified by MOB-typer as IncF, PlasmidFinder also detected IncF replicons in 1058 plasmids (94.6%), but both methods identified different combination of IncF alleles. We observed a high correlation for the presence of IncFII (94.4%), IncFIA (99.2%), and IncFIB (98.3%) alleles. Nevertheless, PlasmidFinder identified the IncFIC allele (AP001918) in 37 plasmids that were not detected by MOB-typer and conversely the allele IncFIIA (AP014877) was identified by MOB-typer in 518 plasmids but not detected by PlasmidFinder. Among the 4359 plasmids that carry unknown replicons (replicon clusters), 3566 were not typed by PlasmidFinder while the other 793 were associated to 128 replicons types.

The comparison of the two replicon databases revealed that MOB-typer shared only 147 sequences with the PlasmidFinder and carry 2336 unique sequences (Supplementary Table S7). The analysis of the 147 shared replicons showed that 10 MOB-typer sequences associated with Inc11, Inc13, and Inc18 were identical to PlasmidFinder sequences that were classified as different types; thus clarifying the divergence mentioned above for Inc11-13-18 plasmids. Similarly for the IncH plasmids, the allele p0111 (AP010962) in PlasmidFinder was identical to IncH (CP021336) in MOB-typer. The comparison also showed that the IncFIIA and IncFIC alleles were unique in MOB-typer and PlasmidFinder, respectively, explaining the different allelic profiles found for IncF plasmids.

### MOB Typing

The second classification method is based on the variation of the relaxase protein. Using the relaxase database of MOB-typer, 821 mobilization protein alleles were detected in 5791 plasmids (47.9%) (Supplementary Table S5). A strong phylum-bias was observed for the detection of relaxases sequences. Indeed, 56.5 and 41.3% of plasmids from *Proteobacteria* and *Firmicutes*, respectively, harbored relaxase proteins but none was identified in *Cyanobacteria*, *Spirochaetes*, or *Tenericutes* (Supplementary Figure S4). While more than 25% of plasmids in the database contained multiple replicons (see above), a single relaxase protein was present in 90% (*n* = 5229) of the plasmids.

Among the 5229 plasmids that could be assigned a MOB type, the most prevalent was MOB_P_ (*n* = 2303) followed by MOB_F_ (*n* = 1422), MOB_Q_ (*n* = 717), MOB_H_ (*n* = 378), MOB_V_ (*n* = 269), and MOB_C_ (*n* = 106). In contrast to these known MOB, 34 relaxases did not belong to the 6 main families but matched sequences classified as “MOB_unknown” from the MOB-typer database. We found that the plasmids harboring a relaxase MOB_C_ or MOB_H_ had a limited host range including 9 and 14 bacterial families, respectively, and were mostly associated with *Enterobacteriaceae* (>80%). MOB_F_ plasmids, found in 43 families, were also highly represented in *Enterobacteriaceae* (67%) whereas the relaxase MOB_V_ were virtually absent in this family. In contrast to the other MOB groups, relaxase belonging to the MOB_P_ and MOB_Q_ were highly promiscuous (BHR plasmids in 80 and 47 bacterial families, respectively) (Figure 2).

Plasmid size trended within MOB types, we observed that 95% of MOB_H_-associated plasmids (*n* = 378) were larger than 110 kb while 91% of MOB_V_ plasmids (*n* = 269) were smaller than 40 kb (Figure 3). The size of the other plasmids (MOB_C_, MOB_P_, and MOB_Q_) was more diversified.

**FIGURE 5:**
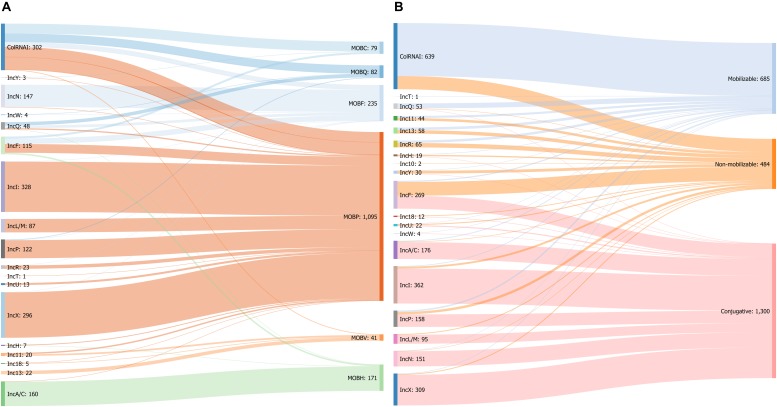
**(A)** Sankey diagram showing the associations between replicon and MOB types in 1703 plasmids. **(B)** Associations between replicon types and predicted mobility in 2469 plasmids from the COMPASS database.

Overall, the analysis of the co-occurrence of the replicons and relaxases in 1703 plasmids revealed that more than 95% of IncI, IncL/M, IncP, IncR, IncT, IncU, IncX, and IncY plasmids were associated with MOB_P_ while IncA/C and IncN plasmids were linked to MOB_H_ and MOB_F_, respectively. In contrast, Inc11, Inc13, and Inc18 plasmids found in *Firmicutes* encoded preferentially MOBv. The other replicon types such as ColRNAI, IncF, and IncQ were not associated with a specific type and carried 4 and 5 different relaxase families (Figure 5A and Table 1).

### Predicted Mobility

Plasmid mobility was predicted based on the presence/absence of three mobilization markers (relaxase, *oriT*, and MPF) (Supplementary Table S5) (Robertson and Nash, 2018).

Using the known *oriT* loci and their matching MOB group from the MOB-typer database, 2048 *oriT* sequences corresponding to the five MOB types present in the database (MOB_C_–associated *oriT* were missing) were detected in 1942 plasmids (16%). The vast majority of plasmids harbored a single *oriT* excepted for 104 plasmids carrying 2 and a single plasmid bearing 3. The most abundant *oriT* loci matched MOB_P_ (*n* = 974) and MOB_F_ (*n* = 482) groups while 96% of the associated plasmids belonged to *Proteobacteria* (*n* = 1624) and *Firmicutes* (*n* = 251). The predicted *oriT*-group matched their relaxase MOB-type for 72% of the plasmids, while discrepancies resulted from the absence of MOB (*n* = 411), a wrong match (*n* = 85), or the detection of an unknown type (*n* = 43).

Finally, 1831 MPF sequences were identified against the MOB-typer database in 4270 plasmids. In comparison to other typing components, the number of MPF per plasmid fluctuated considerably from 1 to 21 genes identified in 608 and 2 plasmids, respectively; 3662 plasmids carried more than 1 MPF and 19% of all MPF-carrying plasmids encoded more than 10 MPFs (Supplementary Figure S7). Regarding the typeability, all the sequences detected within a plasmid were from the same cluster therefore all the 4270 plasmids could be assigned to an MPF type. MPF_T_ and MPF_F_ were the most prevalent types (2053 and 1633 plasmids, respectively) while MPF_I_ was detected in 495 plasmids and MPF_G_ was rare (*n* = 13). Most of the MPF proteins were detected in *Proteobacteria* (86.2%) and *Firmicutes* (9.4%) while no MPF were identified in the phyla *Chlamydiae*, *Cyanobacteria*, *Spirochaetes*, and *Tenericutes*. Specific associations between MPF and replicon types were also observed. MPF_F_ were strongly linked to IncA/C and IncN whereas MPF_T_ and MPF_I_ types were associated with IncX and IncL/M, respectively (Supplementary Figure S8).

Overall, looking at the three dissemination markers, we found that 5882 plasmids (49%) are missing both a relaxase and an *oriT* and were therefore classified as non-mobilizable; 2532 mobilizable plasmids (21%) carrying either a relaxase (*n* = 2121) or an *oriT* (*n* = 218) and 3670 conjugative plasmids (30%) harboring both MOB and MPF.

For the latter plasmids, a strong specificity between four MOB and two MPF types can be observed. Indeed, between 95 and 100% of MOB_C_, MOB_Q_, and MOB_V_ were associated with MPF_T_ while MOB_H_ was linked to MPF_F_. All the MOB_H_ plasmids (*n* = 378) and 82% of MOB_F_ plasmids (*n* = 1175) were predicted to be conjugative whereas 97% of MOB_V_ plasmids were mobilizable.

Regarding the replicon types, IncA/C, IncI, IncL/M, IncN, and IncX plasmids were mostly conjugative (>90% of plasmids predicted to be transferable by conjugation) while none of the ColRNAI, Inc10-11-13, and IncR plasmids were self-transferable. Most of the plasmids belonging to ColRNAI (80%) and IncQ (90%) types were mobilizable. In contrast to these transferable plasmids, 90% of the IncY plasmids were non-mobilizable (Figure 5B and Table 1).

All the plasmids belonging to *Chlamydiae*, *Spirochaetes*, and *Tenericutes*, were predicted to be non-mobilizable whereas the proportion of mobile plasmids (conjugative and mobilizable) varied from 59% in *Proteobacteria* to 15% in *Deinococcus-Thermus* (Supplementary Figure S9). Accordingly, conjugative plasmids were larger (median at 94,307 bp) than non-mobilizable plasmids (median at 36,766 bp), reflecting the genes needed to encode the conjugation functions and the ones conferring selective advantages (Supplementary Figure S10).

## Discussion

### The Comprehensive and Complete Plasmid Database—COMPASS

Plasmids are ubiquitous in bacteria and have been shown to be involved in transferring genes important for survival and fitness to their hosts, enabling rapid adaptation to various selective pressures. They have been widely associated with the global dissemination of antimicrobial resistance and represent an important threat to global health (Chen et al., 2014; Hardiman et al., 2016). Despite the important contribution of plasmids to bacterial evolution, little is known about the global diversity of circulating plasmids and their host range. In order to fill this gap, we curated and compiled all the complete plasmid sequences and associated metadata that were available in the NCBI nucleotide archive to create a comprehensive database.

The initial retrieval of 22,425 plasmid records was followed by several curation steps based on their metadata and nucleotide sequences. The identification of redundant sequences using the CD-HIT-EST tool allowed us to identify clusters of identical plasmids. The analysis of these clusters showed that plasmid dissemination occurs more frequently within the same species (91.8%) rather than transferring horizontally (8.2%) between different species. In addition, these analyses showed that horizontal transmission is favored between species belonging to the same family.

Finally, our new collection composed of 12,084 plasmids covers 1571 distinct bacterial species that were isolated worldwide (126 countries) over more than 100 years (1884–2018). The diversity of the plasmid host range in our database was compared to that of 197,345 bacterial genomes (belonging to 63,240 different Taxonomy IDs) available in the NCBI genome report the 14 April 2019^7^. The host range of the plasmids in our database was found representative of the bacterial genome distribution present in NCBI (i.e., absence of significant differences based on the Kolmogorov–Smirnov test, *p*-value = 0.99). This result partially explained that for many complete plasmid sequences, a complete chromosome sequence from the same isolate may well have been deposited. Among the different bacterial families present in the COMPASS database, we observed an over-representation of plasmids isolated from *Enterobacteriaceae* (*n* = 3963), a family of particular interest for studying antimicrobial resistance as its members have become increasingly resistant to the antibiotics commonly used to treat bacterial infection (Carattoli, 2009; Iredell et al., 2016).

The comparison of COMPASS with previously existing databases showed a similar composition with PLSDB (94%) and pATLAS (82%) in comparison to MOB-suite that only shared 68% with our database. In contrast to the other plasmid resources, our database do not contain any duplicated records, demonstrating the strength of our curation steps.

### Plasmid Sizes and Bacterial Hosts Are Related

Plasmids covered a wide size range and both intra- and inter-phyla variations were observed. Indeed, the smallest (644 bp) and largest (2.5 Mb) plasmids were identified in *Proteobacteria* (*Candidatus Tremblaya* and *Paraburkholderia caribensis*, respectively). This phylum, especially the *Alphaproteobacteria*, harbored 94% of the megaplasmid (>1 Mb). In addition of carrying a large chromosome of several megabases, the presence of megaplasmids is another feature that characterizes *Alphaproteobacteria* (Ettema and Andersson, 2009). In contrast, the smallest plasmids were detected in bacteria belonging to the *Tenericutes* and *Chlamydia* phyla, which also happened to bear the smallest chromosomes (Vetrovsky and Baldrian, 2013; Li and Du, 2014). In our database, almost all chlamydial plasmids (92%) corresponded to an non-conjugative 7.5 kb cryptic plasmid discovered 39 years ago by Lovett et al. (1980) which is involved in the pathogenicity and widely distributed among many different chlamydial strains (Zhong, 2017). These plasmids, although isolated from chlamydial species that infect different animal host species, from different countries and timeline are highly conserved. The lack of diversity of the chlamydial plasmids could be explained by the fact that the members of the *Chlamydia* phylum are a group of obligate intracellular bacteria therefore limiting the propensity for horizontal gene transfer (Blanc et al., 2007).

### Limitations of Current Plasmid Typing Methods

The large number of plasmids in our collection required a thorough classification to decipher plasmid diversity. Here, the *in silico* typing of all the plasmids in our database was performed using the recent reference-based tool MOB-typer (Robertson and Nash, 2018) and the conventional tool PlasmidFinder. MOB*-*typer provides replicon typing similar to PlasmidFinder but with the inclusion of transferability predictions based on the presence of relaxase, oriT, and MPF. Even though several studies investigated the diversity of plasmid mobilization proteins (Garcillán-Barcia et al., 2009, 2011; Fernandez-Lopez et al., 2017), MOB-typer was (at the time of the manuscript writing) the only tool providing *in silico* predictions of the plasmid mobilization system but a novel tool, MOBscan developed by Garcillan-Barcia et al. (2019) recently became available online.

The proportion of plasmids that can be assigned a replicon type is influenced by the dataset and the bioinformatics methods used. Orlek et al. (2017b) found that 85% of the curated *Enterobacteriaceae* plasmids (*n* = 2097) could be replicon-typed (Orlek et al., 2017b). In contrast, only 39.5% of the extensive PLSDB dataset (*n* = 13,789) were typed (Galata et al., 2019). These results both obtained by PlasmidFinder highlight the fact that the performance of the replicon typing is constrained by the bacterial host and most suitable for *Enterobacteriaceae* and Gram-positive species. The percentage of plasmids typed with PlasmidFinder in our study is very similar (40.8%) to the one obtained by Galata et al. (2019). In contrast, MOB-typer was able to type 76% of the plasmids present in COMPASS. Overall, few discrepancies were observed between plasmid assignments of PlasmidFinder and MOB-typer at the nucleotide level (e.g., IncH replicons). However, several plasmid replicons were identified by MOB-typer while they were not detected by PlasmidFinder (e.g., Inc10-11-Inc13-Inc18, IncFIIA, and IncP). The fact that MOB-typer database contains 2021 replicon sequences more than PlasmidFinder and that only 147 sequences are shared between the two databases could explain the observed differences. For example, MOB-typer database contains 34 different replicons associated with IncP while PlasmidFinder possesses only four. In addition, it must be emphasized that 56% of the IncP plasmids undetected by PlasmidFinder are larger than 100,000 bp. IncP plasmids have been reported to be small plasmids (Popowska and Krawczyk-Balska, 2013). However, our analysis showed that the size of IncP plasmid ranges from 3269 and 1,499,175 bp suggesting that some alleles of IncP replicon could be present in larger plasmids than previously reported.

These results highlight the need to fill gaps in plasmid classification, but also the importance of the completeness of plasmid database to have a global view of plasmid properties. With the continuous accumulation of sequencing data, it would be useful to set up a real-time, curated feeding system to maintain comprehensive typing databases. A clear limitation of the current plasmid classification scheme revealed by the present analysis of COMPASS is the frequent occurrence of multiple replicons, complicating the classification.

### Characterization of the COMPASS Database Using MOB-Typer

MOB-typer detected 9231 plasmids bearing replicons in COMPASS. Among these plasmids, 6828 plasmids carried a single replicon of which 2469 belonged to the ColRNAI family and 19 different Inc groups. Unlike replication genes, the presence of multiple relaxases within a plasmid was rare (4%) and the number of unknown relaxases was very low (*n* = 22). Consequently, MOB-typer was able to specifically classified 5207 plasmids into the six MOB families (MOB_C–F–H–P–Q–V_). The most frequent replicon families assigned by MOB-typer were ColRNAI (*n* = 639), IncI (*n* = 362), and IncX (*n* = 309) while the most predominant MOB types were MOB_P_ (*n* = 2303), MOB_F_ (*n* = 1422), and MOB_Q_ (*n* = 717). Overall, we found that half of the plasmids (51%) of the COMPASS database are predicted to be mobilizable or self-transmissible. The percentage of mobile plasmid in our study is higher than the one (39%) found by Smillie et al. (2010) suggesting that new relaxase and MPF sequences were identified in the last decade (Ramachandran et al., 2017).

Our results confirmed that the plasmid typeability differed among the different phyla and we found that plasmids from *Proteobacteria* and *Firmicutes* were the most frequently typed. Our findings regarding the taxonomic diversity of bacterial host among the different Inc groups correlated with previous studies where host ranges are generally narrow for IncF, IncH, IncI, IncT, and IncX or broad for IncA/C, IncP, and IncQ plasmids (Suzuki et al., 2010; Rozwandowicz et al., 2018). ColRNAI have been reported as narrow host range plasmids, i.e., cannot replicate in two different taxonomic classes (Smorawinska et al., 2012). However, the analysis of the COMPASS database revealed the presence of ColRNAI replicons in bacterial species belonging to five different taxonomic classes, although it was mostly found associated to *Gammaproteobacteria* (96%). This result suggests that ColRNAI have a broader replication range than previously reported and should be considered as putative BHR replicon.

Several studies previously showed that IncP plasmids can transfer and replicate in almost all Gram-negative bacteria. These conjugative plasmids are widely present in the environment and are also present in pathogenic and opportunistic bacteria. The presence of antibiotic resistance genes and their broad distribution raises more and more concern (Popowska and Krawczyk-Balska, 2013).

The analysis of COMPASS allowed to conclude at a wide scale on the preferential associations between replicons and relaxases (Garcillán-Barcia et al., 2011; Shintani et al., 2015; Rozwandowicz et al., 2018). IncI, IncL/M, IncP, and IncX replicons were mostly associated with MOB_P_ while IncA/C and IncN were linked to MOB_H_ and MOB_F_. In comparison to these plasmids that were predicted to be mainly conjugative, the replicons belonging to Inc10-11-13-18 were mostly identified on non-mobilizable plasmids harboring MOB_V_. Finally, the BHR ColRNAI and IncQ plasmids were not restricted to one specific relaxase and were mostly mobilizable.

Interestingly, we observed a strong size stratification associated with certain replicon-MOB types: Inc10, Inc11, and Inc13 plasmids carrying MOB_V_ were small (median from 1778 to 3300 bp) while MOB_H_-associated IncA/C plasmids were large (median at 158,754 bp).

### Multireplicon Plasmids Are Widespread Among Circulating Plasmids

Multireplicon plasmids present a particular interest as they had been associated with both virulence and multidrug resistance (Carattoli et al., 2006; Hopkins et al., 2006; Johnson et al., 2007). Multiple replicons were identified in 19% of the characterized plasmids and were represented in all major phyla. Their prevalence could indicate a propensity to circulate between different hosts and thus be vectors more prone to the dissemination of undesirable traits such as antimicrobial resistance and virulence. Indeed, it has already been described that a narrow host range plasmid can broaden its host spectrum by cointegrating a BHR replicon (Osborn et al., 2000; Rozwandowicz et al., 2018). In such multireplicon plasmids, it was suggested that one replicon is expressed due to the selective pressure of plasmid replication while the other is free to diverge. Thus, plasmids can evolve alternatively through replicon acquisition and sequence divergence (Carattoli, 2009; Villa et al., 2010).

Overall, a high degree of plasmid diversity was observed, with 539 different profiles occurring among the 2403 multireplicon plasmids examined. The analysis of these profiles revealed the crucial role of IncF replicon types. These replicons were present in more than half of the multireplicons where they coexist either with other IncF alleles (*n* = 849) or with other Inc groups (*n* = 500). The most frequent replicon associated with IncF was the BHR replicon ColRNAI, thus extending the narrow host spectrum of IncF plasmids.

Multireplicons harboring IncH, IncR, and IncU were also common. Similarly to IncF, these three replicon types were occurring more often in multireplicons (*n* = 159, 106, and 97) than single replicons (*n* = 19, 65, and 22) and the most frequent profile (IncU-IncP) associated a limited host range replicon with a BHR replicon.

### Extensive Diversity of IncF Plasmids and Species-Specific Profiles

IncF plasmids represent one of the most widespread plasmid types in clinical *Enterobacteriaceae* and have been associated with the dissemination of relevant antimicrobial resistance (Johnson et al., 2007; Villa et al., 2010; Rozwandowicz et al., 2018).

Overall, 1118 plasmids (9%) belonging mostly to *Enterobacteriaceae* were classified as IncF plasmids and categorized into single (*n* = 269) and multireplicons (*n* = 849). The occurrence of the four IncF alleles varied between the two categories and the biggest difference was observed for the IncFII replicon, which was present in 19 and 98% of the single and multireplicons, respectively. It has been shown that IncFII are free to diverge when associated with FIA and FIB, evolving toward the formation of new compatible variants (Villa et al., 2010; Rozwandowicz et al., 2018). Thus, the acquisition and the sequence divergence of IncFII enable the replication in a broader host spectrum and contribute to the global dissemination of IncF. Preferential associations between bacterial hosts and IncF profiles were observed. For instance, *K. pneumoniae* and *S. enterica* both carried favorably IncFIB and IncFII in single replicons and these two alleles also coexisted in multireplicons suggesting that these plasmids could have arisen from recombination of two single IncF plasmids. Regarding the mobilization system, our results correlate with previous studies (Rozwandowicz et al., 2018). While 68% were predicted to be conjugative, 82 and 85% of the IncF plasmids were associated with MOB_F_ and MPF_F_.

## Conclusion

It has been shown that highly similar bacterial strains can contain a compelling variety of different plasmids (Lanza et al., 2014). The analysis of the diversity and host range of circulating plasmids is therefore essential to characterize bacterial isolates. Overall, *in silico* typing is likely to remain an important tool for plasmid analysis, but their performance is constrained by the diverse and dynamic nature of plasmids, the plasmid dataset of interest, and the applied bioinformatics methods. We showed the advantages of using MOB-typer versus PlasmidFinder that currently does not characterize the mobility of plasmids and only includes replicons from *Enterobacteriaceae* and a few Gram-positive bacteria. However, more efforts need to be done to develop a general classification scheme for plasmids from all microbial lineages. COMPASS plasmids could be easily integrated as a reference database into pipelines for reconstructing plasmids and ascertaining host range properties to new identified plasmids. The complete classification will help deciphering the molecular epidemiology of antimicrobial resistance by identifying specific types and plasmid features linked to a particular resistance gene.

## Data Availability Statement

The plasmid sequences of the COMPASS database compiled in this project, the metadata associated for each plasmid, and the HTML link for the interactive Krona chart are available online on the following GitHub repository: https://github.com/itsmeludo/COMPASS.

## Author Contributions

M-YM conceived and piloted the project. P-ED carried out the analyses and wrote the manuscript. LM developed the script for the creation and curation of the database. LM, NR, and AF provided advice and help for bioinformatics and genome analyses. M-YM, NR, LM, and AF participated in the discussion and reviewed the manuscript.

## Conflict of Interest

The authors declare that the research was conducted in the absence of any commercial or financial relationships that could be construed as a potential conflict of interest.

## Footnotes

1 https://pubmlst.org/rmlst/

2 https://github.com/phac-nml/mob-suite

3 https://cge.cbs.dtu.dk/services/PlasmidFinder/

4 http://lert.co.nz/map/

5 http://jvenn.toulouse.inra.fr/app/index.html

6 http://sankeymatic.com/

7 ftp://ftp.ncbi.nlm.nih.gov/genomes/GENOME_REPORTS/

## Supplementary Material

FIGURE S1 Network of identical plasmids isolated across different bacterial species from the COMPASS database (*n* = 54). Each line represents an identical plasmid shared between two bacterial species and the thickness of the line represent the number of plasmid cluster. Each color represents a bacterial family.

FIGURE S2 Comparison of the plasmid contents of four plasmid databases (COMPASS, PLSDB, pATLAS, and MOB-suite).

FIGURE S3 World map and histogram showing the distribution and the occurrence of plasmids isolated per countries and per year.

FIGURE S4 Detection of plasmid replicon and relaxase (in%) among the different phyla from the COMPASS database (*n* = 12,084). The phylum entitled “Other” is composed of 57 plasmids from 12 minority phyla (*n* < 25) and 129 plasmids missing taxonomy data.

FIGURE S5 Correlation between the number of replicons and the plasmid size from the COMPASS database (*n* = 9231).

FIGURE S6 Network of multiple replicon types (i.e., >2) isolated from different bacterial species from the COMPASS database (*n* = 1349). Each line represents shared replicon types harbored by plasmids and the thickness of the line represents the number of replicons types harbored by these latter.

FIGURE S7 Number of mating pore formation (MPF) proteins per plasmids.

FIGURE S8 Sankey diagram showing the associations between replicon and MPF types in 1 378 plasmids from the COMPASS database.

FIGURE S9 Percentage of transferable and non-mobilizable plasmids among the different phyla from the COMPASS database (*n* = 12,084).

FIGURE S10 Violin plot displaying plasmid size distribution (log_10_) among non-mobilizable and transferable plasmids from the COMPASS database (*n* = 12,084).

TABLE S1 Curation of COMPASS database.

TABLE S2 CD-HIT clusters analysis.

TABLE S3 Comparison of plasmid databases.

TABLE S4 Description of COMPASS metadata.

TABLE S5 MOB-typer results.

TABLE S6 Comparison of PlamidFinder and MOB-typer results.

TABLE S7 Comparison of plasmidFinder and MOB-typer replicon databases.
