# Supplementary figures and images for: Analysis of COMPASS, a New Comprehensive Plasmid Database Revealed Prevalence of Multireplicon and Extensive Diversity of IncF Plasmids
Source: Front Microbiol. 2020 Mar 24;11:483. doi: 10.3389/fmicb.2020.00483 (PMC7105883; doi:10.3389/fmicb.2020.00483)

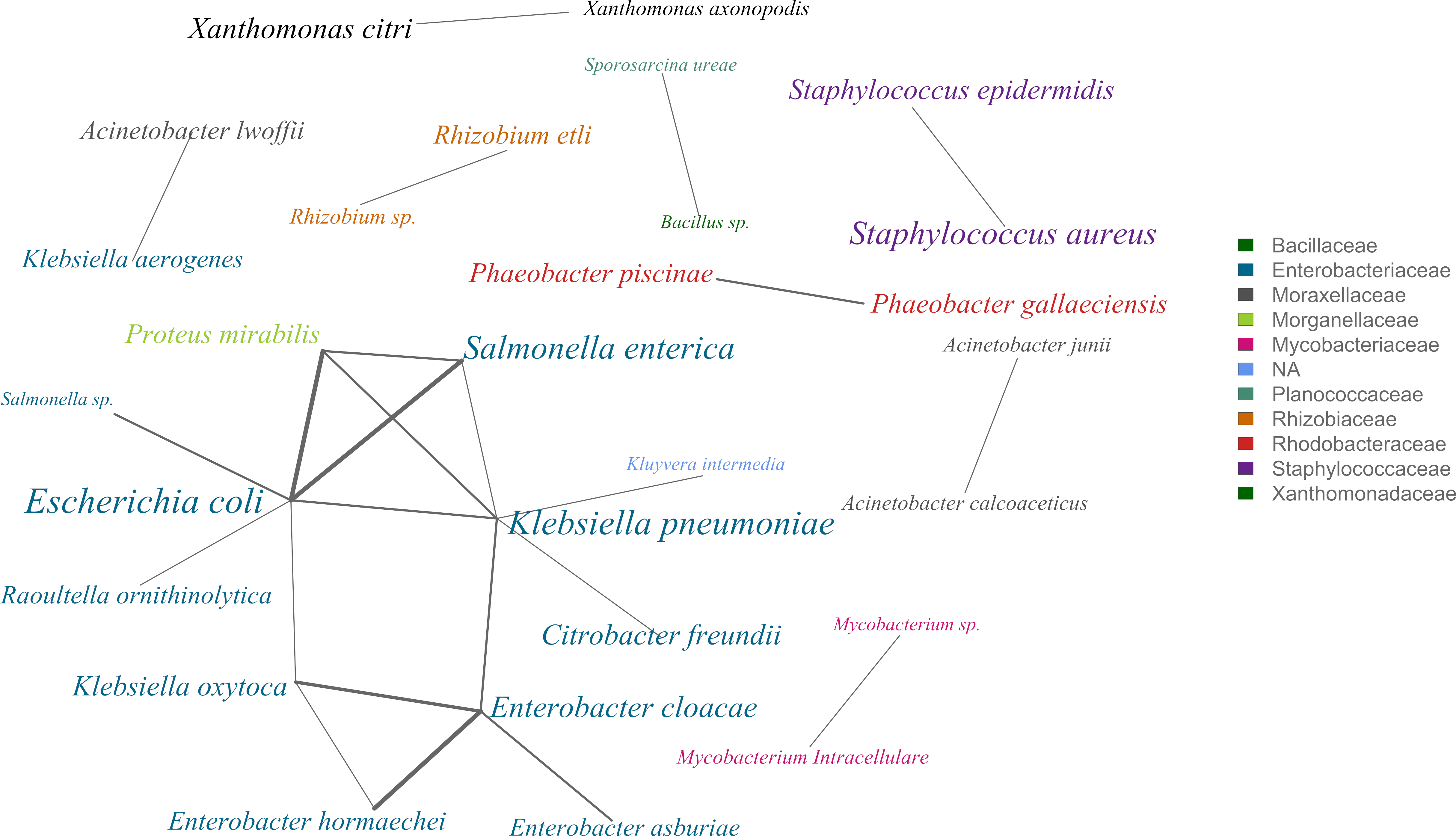

Supplement: FIGURE S1 — Network of identical plasmids isolated across different bacterial species from the COMPASS database (n = 54). Each line represents an identical plasmid shared between two bacterial species and the thickness of the line represent the number of plasmid cluster. Each color represents a bacterial family. [file Image_1.TIF]

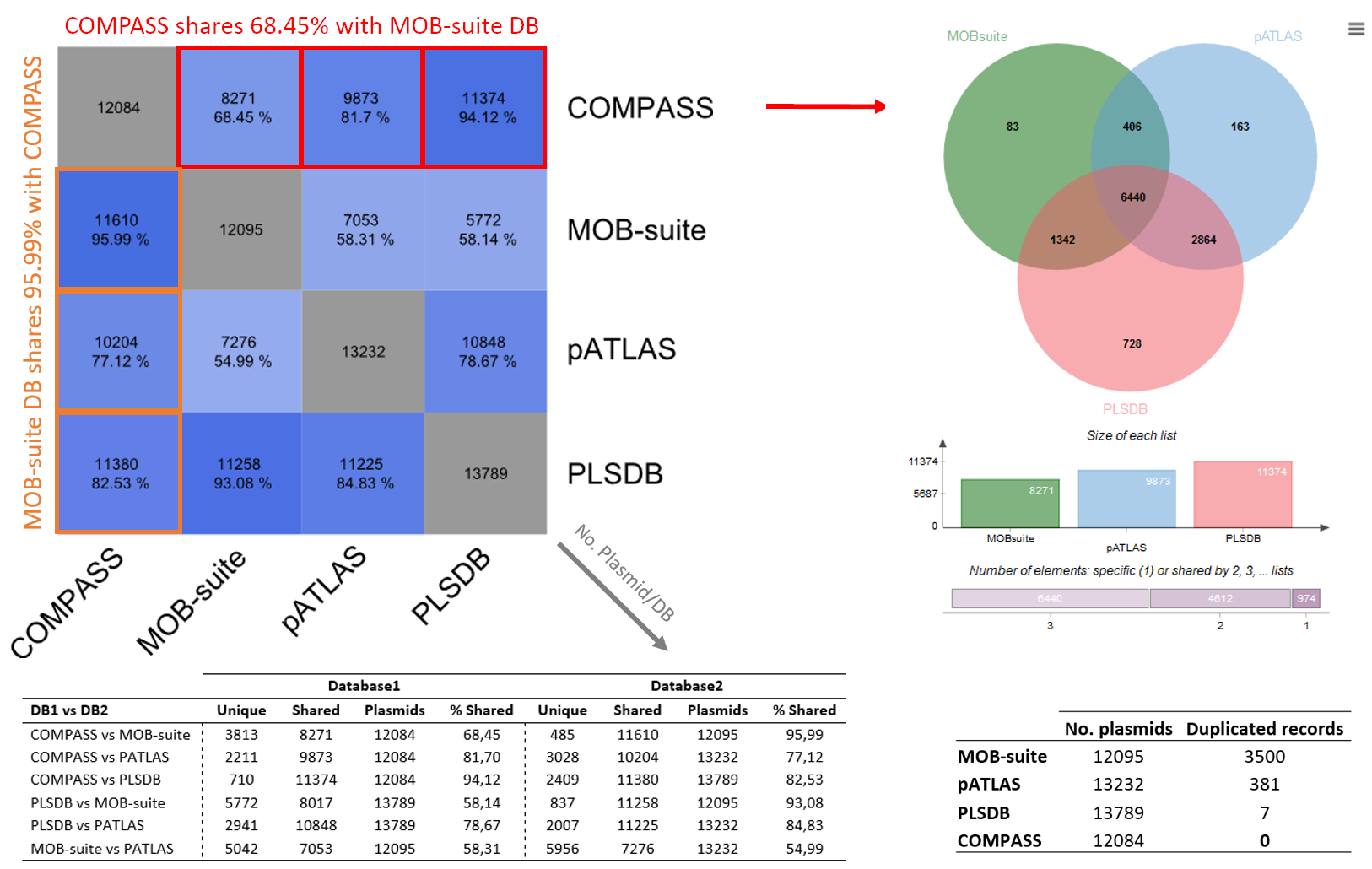

Supplement: FIGURE S2 — Comparison of the plasmid contents of four plasmid databases (COMPASS, PLSDB, pATLAS, and MOB-suite). [file Image_2.TIF]

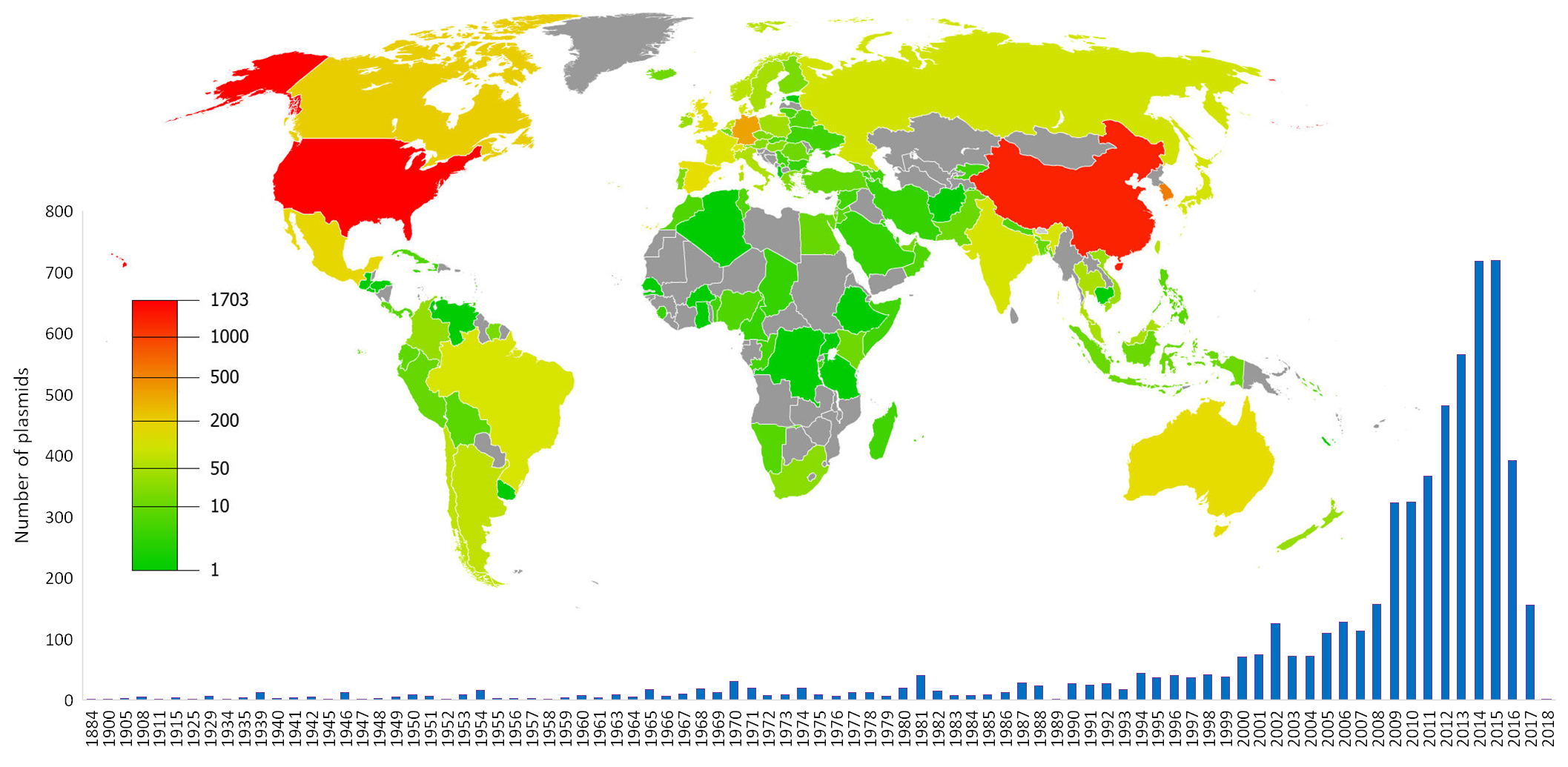

Supplement: FIGURE S3 — World map and histogram showing the distribution and the occurrence of plasmids isolated per countries and per year. [file Image_3.TIF]

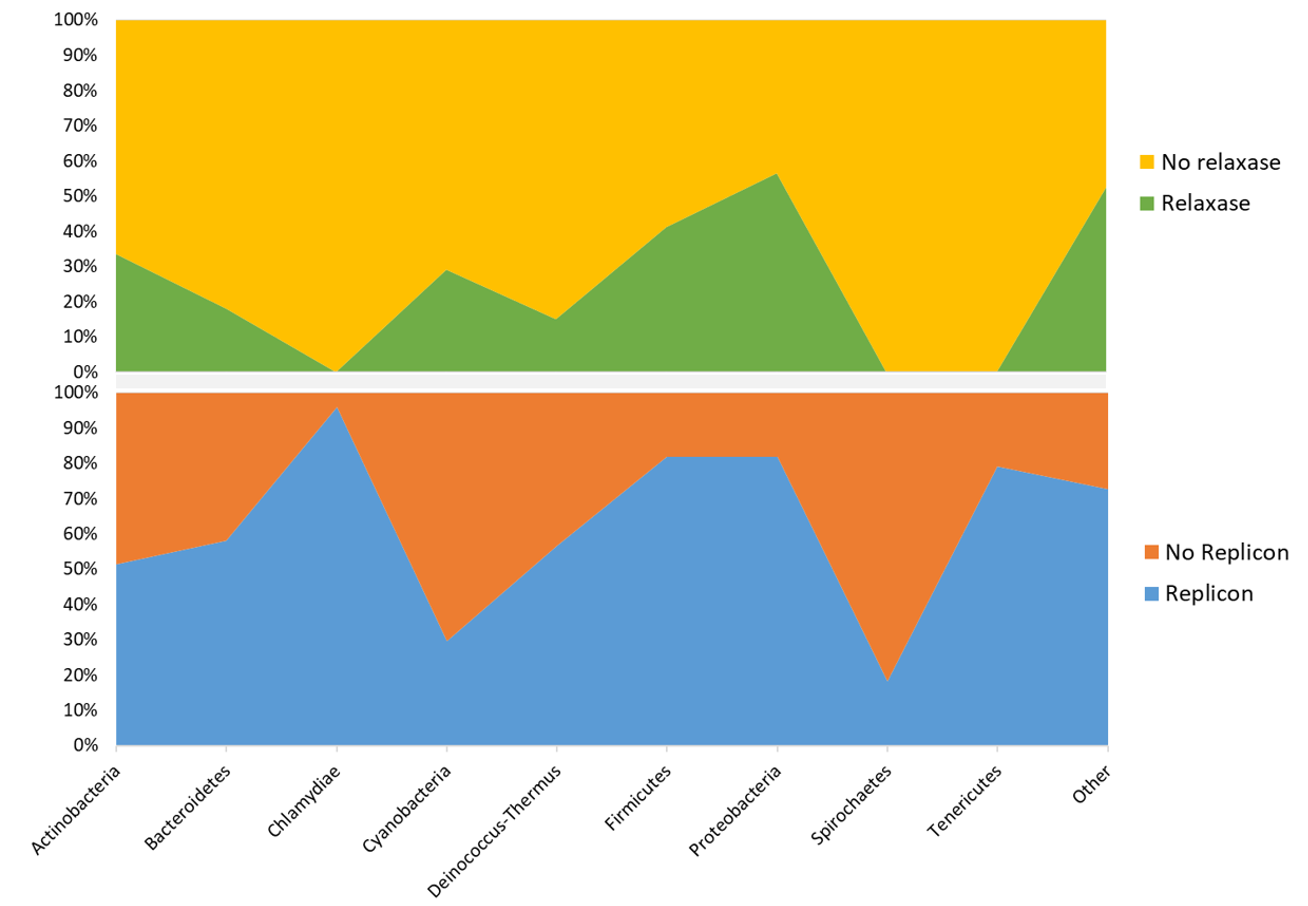

Supplement: FIGURE S4 — Detection of plasmid replicon and relaxase (in%) among the different phyla from the COMPASS database (n = 12,084). The phylum entitled “Other” is composed of 57 plasmids from 12 minority phyla (n < 25) and 129 plasmids missing taxonomy data. [file Image_4.TIF]

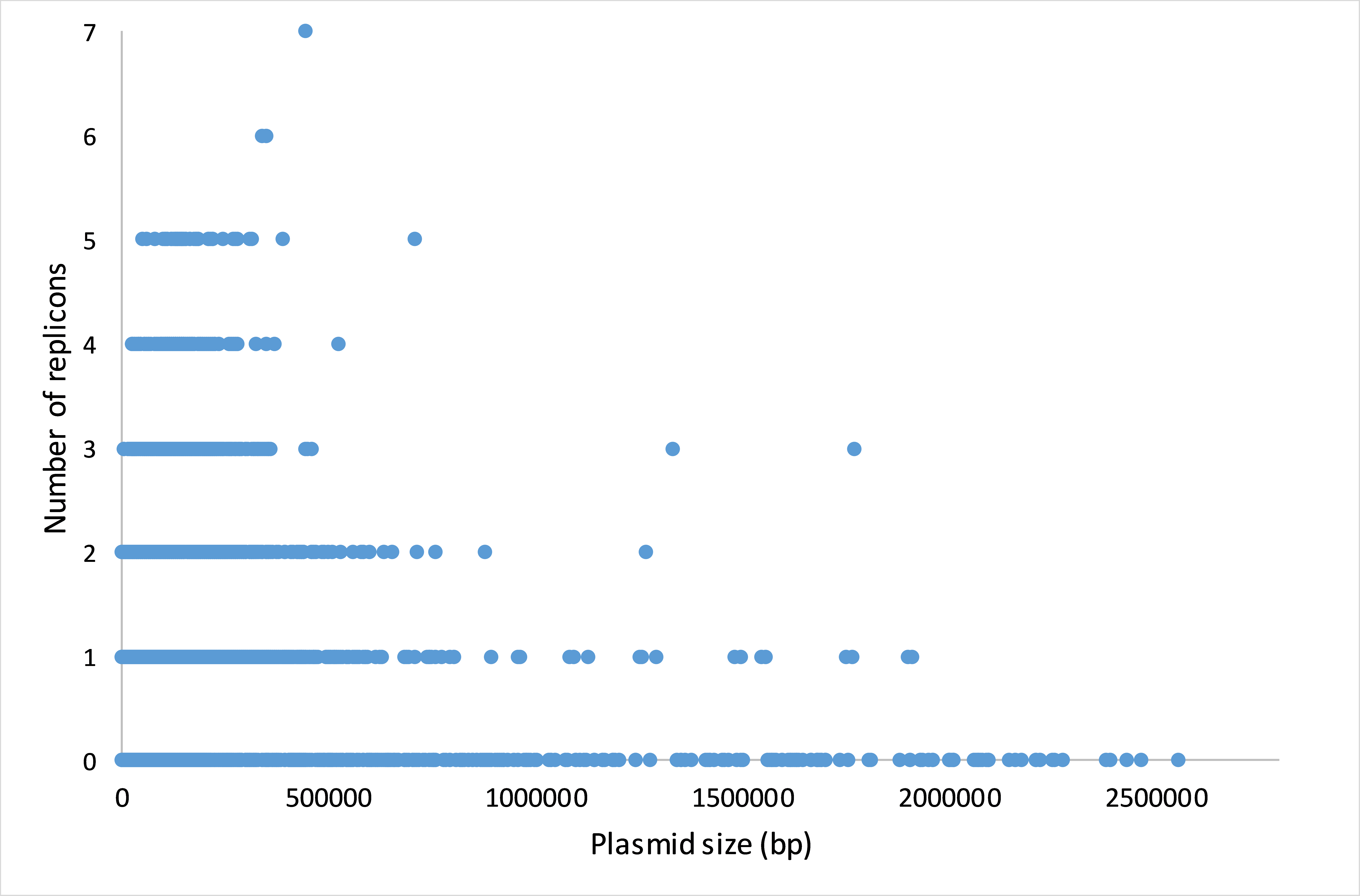

Supplement: FIGURE S5 — Correlation between the number of replicons and the plasmid size from the COMPASS database (n = 9231). [file Image_5.TIF]

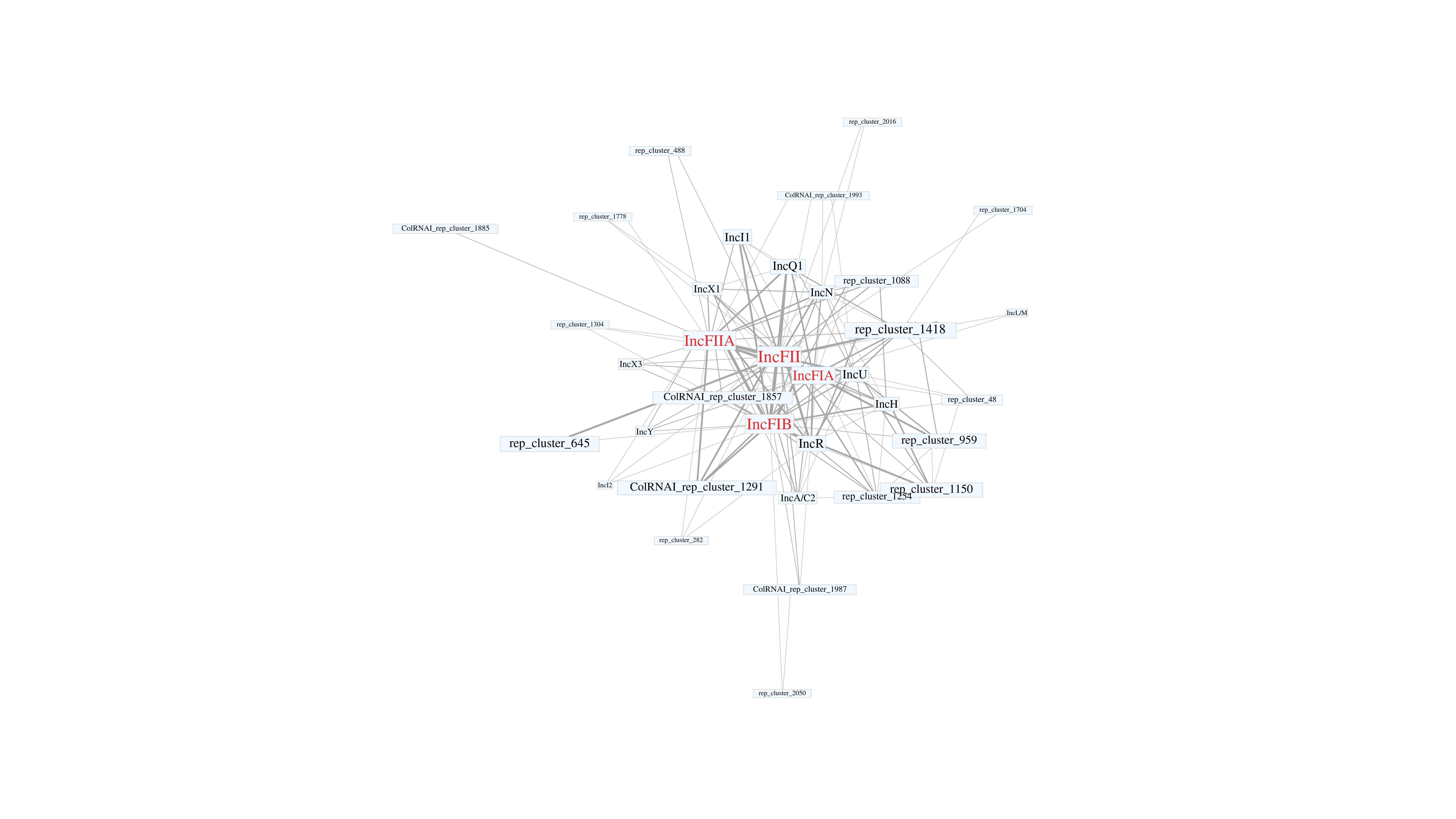

Supplement: FIGURE S6 — Network of multiple replicon types (i.e., >2) isolated from different bacterial species from the COMPASS database (n = 1349). Each line represents shared replicon types harbored by plasmids and the thickness of the line represents the number of replicons types harbored by these latter. [file Image_6.TIF]

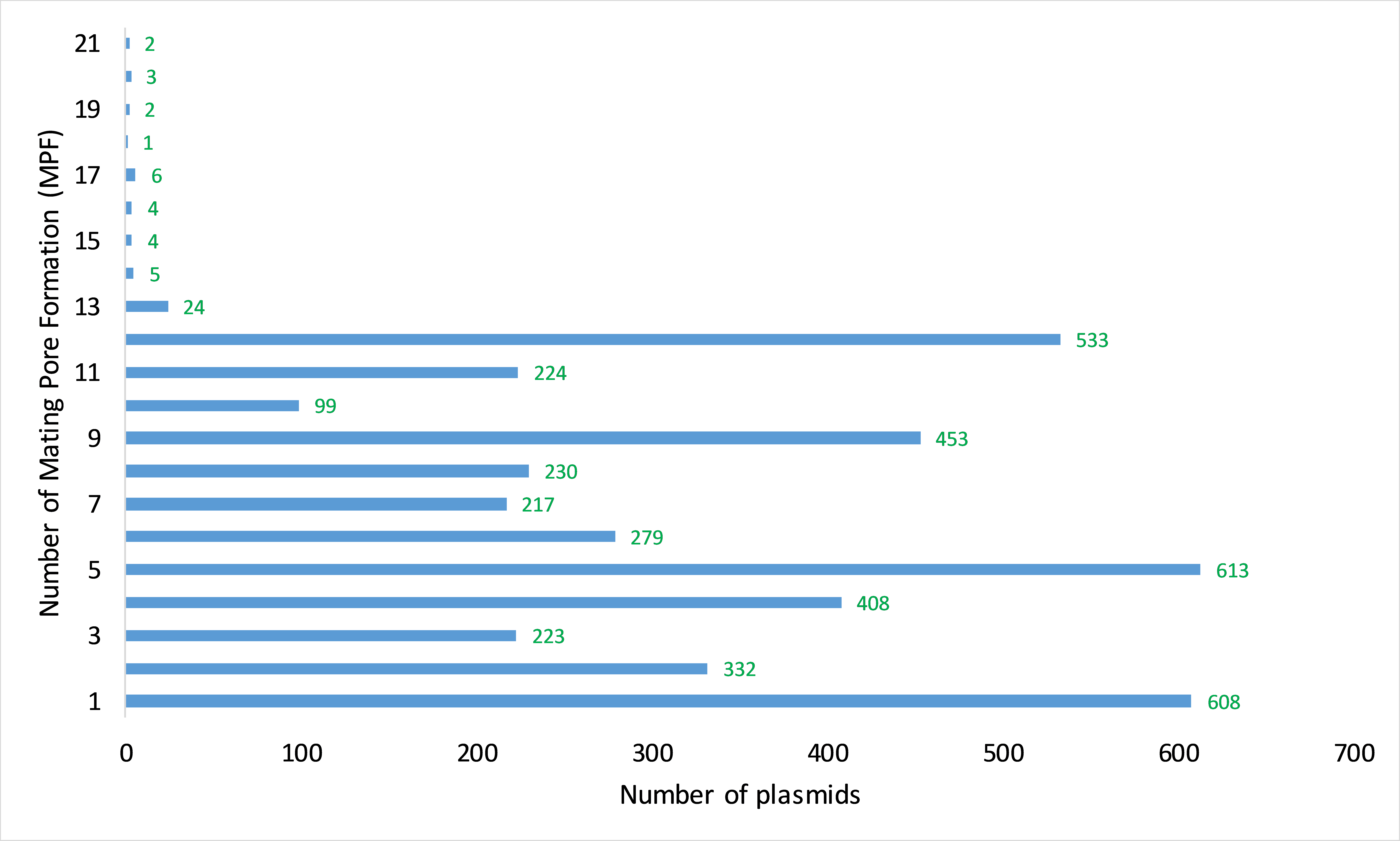

Supplement: FIGURE S7 — Number of mating pore formation (MPF) proteins per plasmids. [file Image_7.TIF]

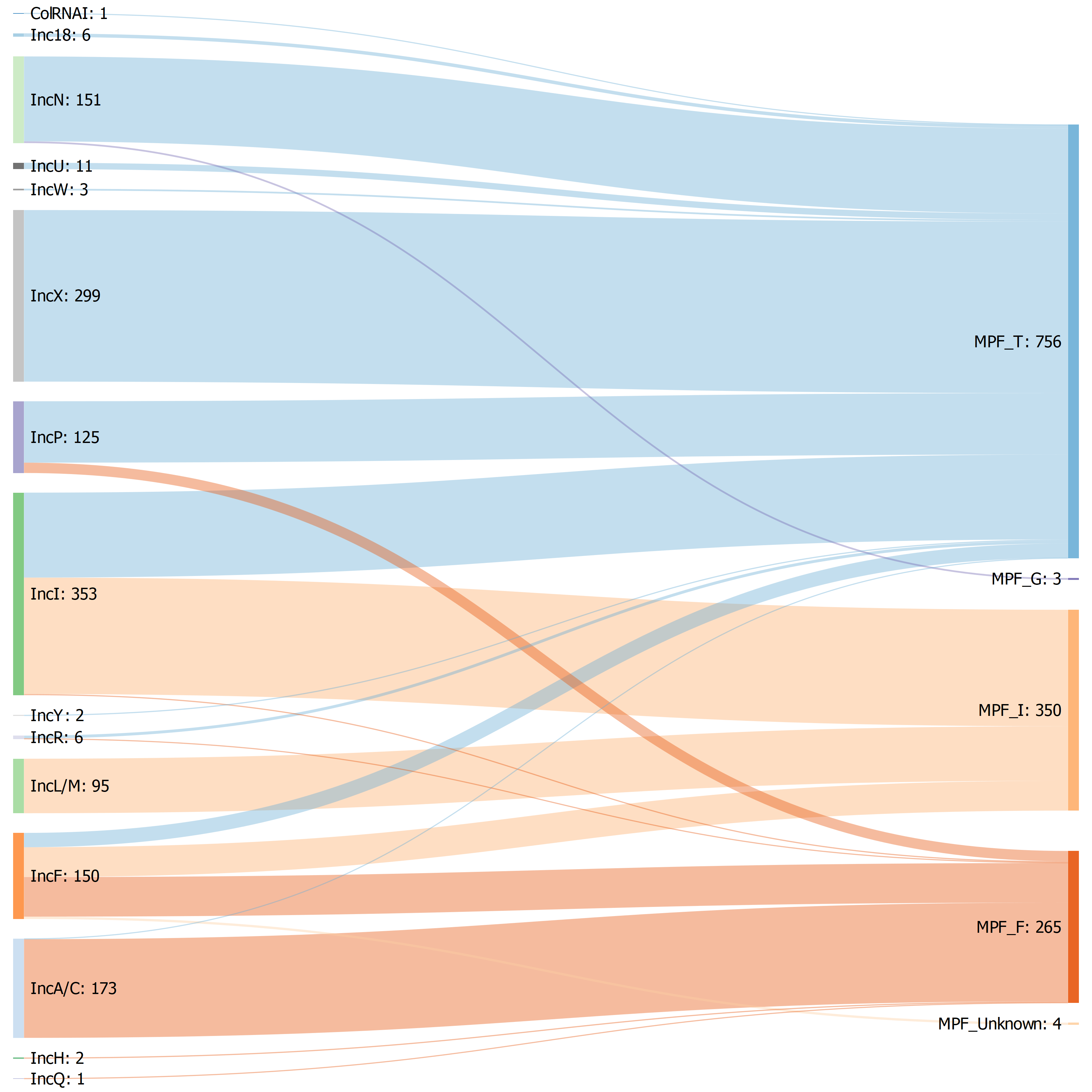

Supplement: FIGURE S8 — Sankey diagram showing the associations between replicon and MPF types in 1 378 plasmids from the COMPASS database. [file Image_8.TIF]

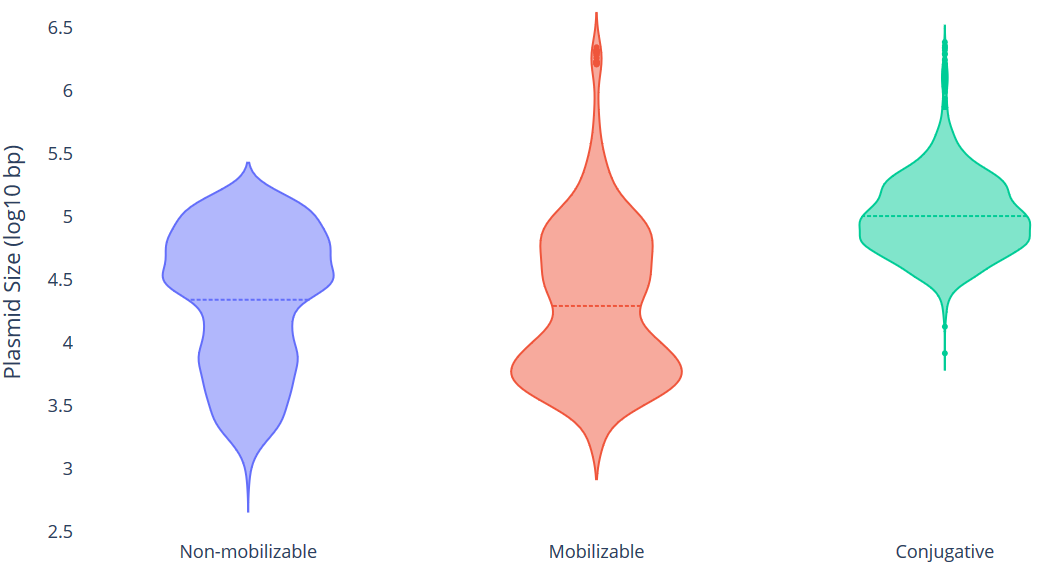

Supplement: FIGURE S10 — Violin plot displaying plasmid size distribution (log10) among non-mobilizable and transferable plasmids from the COMPASS database (n = 12,084). [file Image_10.TIF]
